# Airway Hyperresponsiveness to Mannitol in Obesity Before and After Bariatric Surgery

**DOI:** 10.1007/s11695-014-1564-8

**Published:** 2015-01-25

**Authors:** Ebymar Arismendi, Eva Rivas, Josep Vidal, Esther Barreiro, Yolanda Torralba, Felip Burgos, Roberto Rodriguez-Roisin

**Affiliations:** 1 Servei de Pneumologia (Institut del Tòrax), and Fundació Clínic per la Recerca Biomèdica, Hospital Clínic, Barcelona, Spain; 2 Servei de Anestesiologia and Fundació Clínic per la Recerca Biomèdica, Hospital Clínic, Barcelona, Spain; 3 Institut d’Investigacions Biomédiques August Pi i Sunyer (IDIBAPS), Universitat de Barcelona, Barcelona, Spain; 4 Ciber Enfermedades Respiratorias (CIBERES), Barcelona, Spain; 5 Servei de Endocrinologia, and Fundació Clínic per la Recerca Biomèdica, Hospital Clínic, Barcelona, Spain; 6 Pulmonology Department, Hospital del Mar, Parc de Recerca Biomèdica de Barcelona (PRBB), Universitat Pompeu Fabra, Barcelona, Spain; 7 Servei de Pneumologia (Institut del Tòrax), and Fundació Clínic per la Recerca Biomèdica, Ciber Enfermedades Respiratorias (CIBERES), Barcelona, Spain; 8 Institut Clínic del Tòrax, Servei de Pneumologia, Hospital Clínic, Villarroel, 170, Barcelona, 08036 Spain; 9 Ciber Diabetes y Enfermedades Metabólicas (CIBERDEM), Barcelona, Spain

**Keywords:** Abdominal obesity, Airway inflammation, Bronchial hyperreactivity, Indirect bronchoconstrictor, Systemic inflammation

## Abstract

**Background:**

The relationship between airway hyperresponsiveness (AHR) and obesity, a low-grade systemic inflammatory condition, remains largely unknown. It is established that AHR to indirect stimuli is associated with active airway inflammation. The objectives were to investigate the rate of AHR to mannitol in obese subjects and its changes 1 year after bariatric surgery (BS).

**Methods:**

We enrolled 58 candidates to BS severely obese (33 nonsmokers and 25 smokers) without history of asthma and 20 healthy, nonobese participants and related AHR to functional findings and serum and exhaled biomarkers.

**Results:**

Before surgery, AHR was observed in 16 (28 %) obese with the provocation doses of mannitol to induce a 15 % fall in FEV_1_ (PD_15_) of (geometric mean [95 % CI]) 83 (24–145) mg. Compared to control participants, obese participants had lower spirometric values and higher serum and exhaled biomarkers (*p* < 0.05 each). After surgery, AHR was abolished (*p* < 0.01) in all but four obese subjects.

**Conclusions:**

Weight loss induced by BS was the key independent factor associated to AHR improvement. AHR to mannitol is highly prevalent in obesity, and it is largely abolished by BS.

## Introduction

The concurrence of obesity and bronchial asthma has become an increasing worldwide major public health problem [1, 2]. Some studies show a clear evidence for a relation of airway hyperresponsiveness (AHR) to body mass index (BMI) [3]. However, the mechanisms of the relationship between obesity, AHR, and asthma are not sufficiently established [4, 5], and the impact of its underlying low-grade chronic systemic inflammation on AHR remains unsettled [6]. Obesity is characterized by an increased number of resident macrophages in adipose tissue that secrete a variety of inflammatory molecules, such as leptin and adiponectin [7, 8]. It is known that AHR to osmotic challenge agents is associated with the presence of airway inflammation as they increase airway osmolarity and induce the release of inflammatory cells and of mediators that act on their specific receptors on the airway smooth muscle causing contraction [9, 10].

Bariatric surgery (BS) is the most efficacious therapeutic strategy to achieve major and sustained weight loss in morbidly obese subjects [11] and is associated with improved systemic inflammation [12]. There have been very few studies in obese subjects, mostly in asthmatics, to assess AHR using methacholine [13] before and after effective weight loss with discrepant results [14–17]. We investigated the prevalence of AHR to mannitol in obese candidates to BS before and 1 year after BS. Part of the results of this study has been previously reported in abstract form [18].

## Methods

### Subjects

This is a prospective, observational study in 58 morbidly obese individuals (44 females/14 males; aged 46 ± 12 years) with BMI ≥40 kg/m^2^ or ≥35 kg/m^2^ (in patients with obesity-related diseases), candidates to BS in our center [18]. This sample of obese subjects was part of a study with a larger cohort of severe obese candidates to BS aimed to describe the systemic inflammome before and after BS [19]. Thirty-three obese individuals were nonsmokers (<10 pack-years) and 25 current (≥10 pack-years) or former (≥1 year after complete cessation) smokers (38 ± 26 pack-years). Participants with cardiovascular and pulmonary (i.e., asthma, chronic obstructive pulmonary disease, and bronchiectasis) diseases were excluded. One year after BS, participants were also evaluated. A control group of 20 healthy nonsmokers (16 females/4 males), with normal weight and lung function, age-matched (46 ± 7 years) was also recruited. Written informed consent (Protocol 2008/4015) was obtained from each participant according to the requirements of the Ethics Committee of the *Hospital Clínic*, *Universitat de Barcelona*.

BS including either Roux-en-Y gastric bypass or sleeve gastrectomy was carried out according to standard procedures [20]. The selection of the surgical technique was based on the presence of larger BMI, an estimated operative risk, or the presence of an enlarged liver [21]. Postoperative weight loss was expressed as a percentage of the presurgical excess weight (% excess weight loss = [100 × [weight before surgery − weight at the time of evaluation] / [weight before surgery − weight corresponding to body mass index = 25 kg/m^2^]) [20]. BS was considered successful when excess weight loss was >50 % of presurgical excess weight.

### Study Design

The 58 enrolled obese subjects visited the laboratory twice on two consecutive days, before (median, 5 weeks) and 1 year after BS (median, 13 months). At day 1, clinical evaluation, forced spirometry, and static lung volumes using our own predicted values [22–25] were carried out; at day 2, serum and exhaled samples were obtained, and the mannitol challenge was performed in all obese participants. At day 1, serum and exhaled samples and forced spirometry were determined in all control individuals; at day 2, eight subjects were challenged to mannitol alone. Control participants were explored once only.

### Mannitol Challenge

Mannitol challenge was carried out using a commercially available kit (Pharmaxis Ltd, Burnham, UK) [26]. Bronchial challenge to mannitol was performed as previously reported by our group [27]. Pre-challenge baseline FEV_1_ was used to calculate the maximum % fall FEV_1_ after mannitol. The dose of mannitol that induced a fall of 15 % below baseline (i.e., PD_15_) was expressed as geometric mean (GM) (GM PD_15_) in milligrams. AHR to mannitol was defined as PD_15_ < 635 mg. The response-dose ratio (RDR) was calculated as the % fall FEV_1_ at the last dose, divided by the total cumulative dose of mannitol given (expressed as % fall/mg) in milligrams administered [26]. The test stopped when 15 % fall FEV_1_ was achieved or the cumulative dose of 635 mg had been administered. Mannitol bronchoprovocation was completed by control and obese individuals without major adverse events.

### Biomarker Measurements

Serum and exhaled samples were obtained and stored at −80 °C. Serum concentrations of C-reactive protein (CRP) were determined using an immunoturbidimetry method (ADVIA Chemistry, Siemens Tarrytown, NY, USA) and those of leptin using the specific enzyme-linked immunoabsorbent assay (ELISA) kit (Diagnostic Biochem Canada Inc., Ontario, Canada). Serum adiponectin, soluble tumor necrosis factor-receptor 1(sTNF-R1), and interleukin (IL)-8 levels were determined using a specific ELISA (US Biological Salem, MA, USA; IBL international Hamburg, Germany; and Anogen Ontario, Canada), respectively. Exhaled breath condensate samples of IL-8 were determined using an EcoScreen condenser (Jaeger, Würzburg, Germany) following current recommendations [28, 29] and specific ELISA kits (Cayman Chemical Company, Ann Arbor, MI, USA).

All biomarker measurements were performed in duplicate and the mean value used for analysis. Biomarker concentrations were below the lower limit of quantification (LLQ) in some individuals. To avoid a downward bias of biomarkers, a nominal level of half of the LLQ value was used in the analysis in individuals with values below the LLQ [30].

### Statistical Analysis

Data were expressed as mean ± SD or median (interquartile range). Comparisons between obese and control individuals before BS were evaluated using one-way ANOVA with Bonferroni post hoc analysis (parametric) and Kruskal-Wallis (nonparametric) test. For comparisons between obese subjects before and after surgery, paired Student’s for related samples (parametric) or Wilcoxon (nonparametric) tests for continuous variables were used. Unpaired Student’s (parametric) and Mann-Whitney *U* (nonparametric) tests with Bonferroni-Holm correction were performed to compare obese individuals with and without AHR. Chi-square and McNemar tests for categorical variables and Spearman correlations were used as appropriate. An odds ratio calculated using generalized estimating equations (GEE) analysis [31], to account for non-independent evaluations from the same individuals before and after BS, was determined to assess the potential relationships of anthropometric data, cigarette smoking, and serum and exhaled biomarkers with AHR response. Statistical analysis was performed with specialized computer software (SPSS 20.0, Chicago, IL). Unless otherwise stated, significance was set at *p* < 0.05.

## Results

### Baseline Findings (Before Bariatric Surgery)

The most relevant characteristics of obese and control individuals are displayed in Table 1. Obese participants had, compared to control, decreased spirometric values, higher FEV_1_/FVC ratios, and higher serum and exhaled biomarkers but lower adiponectin levels. In obese subjects, comorbidities were common. Eight obese nonsmokers (14 % of total and 24 % of 33 nonsmokers) (median cumulative dose of inhaled mannitol, 176 mg [range, 5–629 mg]) and the same number of obese smokers (8 [14 % of total and 32 % of 25 smokers]) (121 mg [range, 5–475 mg]), respectively, were responsive to mannitol. Responsive obese nonsmokers had, compared to responsive smokers, lower mannitol-induced baseline FEV_1_ (2.59 ± 0.63 and 3.15 ± 0.65 l) and final FEV_1_ (1.88 ± 0.56 and 2.57 ± 0.64 l) values, respectively (*p* < 0.05 each), without differences in the rates of AHR (chi-square test) (Table 2). The higher were the exhaled IL-8 levels in obese individuals, the greater were reactivity (RDR) (Rho, 0.35, *p* < 0.01) and % fall FEV_1_ (Rho, 0.32, *p* < 0.05) to mannitol. AHR was not documented in any of the eight control subjects. There were no differences between obese nonsmokers and smokers who completed the study.

**Table 1:** Demographic, clinical, and functional characteristics and serum and exhaled biomarkers in control and obese participants before and after bariatric surgery

	Control subjects (*n*, 20)	Obese subjects (*n*, 58)		*p* value
		Before	After	
Age, years	46 ± 7	46 ± 12	47 ± 12	–
Female, *n* (%)	16 (80)	44 (76)	–	–
BMI, kg/m^2^	22 ± 3 *	45 ± 5 *	30 ± 4	<0.001
Waist circumference, cm	81 ± 11 *	130 ± 11 *	97 ± 11	<0.001
OSA, *n* (%)	–	41 (71)	12 (21)	<0.001
MS, *n* (%)	0	44 (76)	10 (17)	<0.001
FVC, % pred	105 ± 12*	90 ± 13*	101 ± 13	<0.001
FEV_1,_ % pred	103 ± 13**	94 ± 15**	102 ± 13	<0.001
FEV_1_/FVC, %	78 ± 5*	83 ± 5 *	80 ± 5	<0.001
BD response, %	2 ± 3	5 ± 6	2 ± 4	<0.01
FRC, % pred	ND	73 ± 14	112 ± 22	<0.001
ERV, % pred	ND	39 ± 25	113 ± 32	<0.001
C-reactive protein, mg/l	0.4 (0.1–0.5)*	6.4 (3.7–11.4)*	0.4 (0.2–0.8)	<0.001
Leptin, ng/ml	13.6 (5.6–19.7)*	46.3 (38.4–96.9)*	13.6 (6.9–22.1)	<0.001
Adiponectin, μg/ml	22.1 (18.1–25.9)*	13.4 (8.7–18.8)*	19.0 (12.0–25.2)	<0.001
sTNF-R1, ng/ml	0.4 (0.2–0.5)*	1.4 (1.0–1.9)*	0.9 (0.3–1.3)	<0.001
IL-8, pg/ml	2.0 (2.0–3.3)	9.4 (2.0–31.7)	3.4 (1.9–14.2)	0.16
Exhaled IL-8, pg/ml	0.9 (0.4–4.0)**	4.4 (1.5–6.6)**	4.0 (1.1–7.1)	0.62

Unless otherwise stated, data are expressed as mean ± SD, *n* (%) or median (interquartile range)

*BMI* body mass index, *OSA* obstructive sleep apnea, *MS* metabolic syndrome, *pred* predicted, *BD* bronchodilator, *FVC* forced vital capacity, *FEV*_*1*_ forced expiratory volume in the first second, *FRC* functional residual capacity, *ERV* expiratory reserve volume, *sTNF-R1* soluble tumor necrosis factor-receptor 1, *IL* interleukin

*p < 0.001 for comparisons between control and obese subjects before surgery; **p < 0.05 for comparisons between control and obese subjects before surgery

**Table 2:** Differences in outcomes between obese subjects with and without airway hyperresponsiveness (AHR) to mannitol before and after bariatric surgery

	Before		*p* value^a^	After		*p* value^b^
	With AHR	Without AHR		With AHR	Without AHR	
Patients, *n*	16	42	–	4	54	–
Age, years	49 ± 7	46 ± 12	0.29	60 ± 4	46 ± 10	0.009
BMI, kg/m^2^	46 ± 5	44 ± 5	0.79	27 ± 3	30 ± 4	0.11
Waist, cm	134 ± 9	128 ± 11	0.052	97 ± 11	97 ± 11	0.95
Baseline FEV_1_, % pred	89 ± 15*	96 ± 15	0.15	117 ± 23**	99 ± 13	0.014
Final FEV_1_, % pred	70 ± 18*	91 ± 18	<0.001	97 ± 17**	94 ± 14	0.79
% Fall FEV_1_	21 ± 9	6 ± 4	<0.001	16 ± 1	3 ± 6	<0.001
GM PD_15_, mg	83 (24–145)	>635	NA	224 (156–322)	>635	NA
RDR, %/mg	0.11 (0.05–1.88)	0.007 (0.003–0.129)	<0.001	0.05 (0.05–0.17)	0.008 (0.003–0.011)	<0.001
Exhaled IL-8, pg/ml	6.23 (2.94–12.66)	3.97 (1.26–5.70)	0.99	7.10 (2.58–11.46)	3.90 (1.07–6.51)	0.44

Unless otherwise stated, data are expressed as mean ± SD or median (interquartile range); baseline FEV_1_, FEV_1_ values measured after inhaling the 0-mg of mannitol capsule; final FEV_1_, FEV_1_ values measured at the end of mannitol test; % fall FEV_1_, mannitol-induced percentage decrease of FEV_1_ from baseline to final values; GM (geometric mean) of PD_15_, provocation dose of mannitol to cause a 15 % fall FEV_1_; RDR, response-dose ratio (% fall FEV_1_ divided by cumulative dose given of mannitol). For other abbreviations, see Table 1

*p < 0.001 for comparisons between baseline and final FEV_1_ values before surgery; **p < 0.05 for comparisons between baseline and final FEV_1_ values after surgery

^a^ *p* value for comparisons between obese subjects with and without AHR before surgery

^b^ *p* value for comparisons between obese subjects with and without AHR after surgery

The logistic regression model fitted to data for all obese individuals did not yield interaction (all *p* values >0.4), without significant associations with anthropometric co-variables or cigarette smoking. The levels of exhaled IL-8 had a marginal association with AHR (odds ratio [OR], 1.10; 95 % confidence interval [CI], 0.97–1.22; *p* = 0.06) only.

### Findings after Bariatric Surgery

Sleeve gastrectomy (SG) was performed in 32 and Roux-en-Y gastric bypass (RYGBP) in 26 subjects, without differences in the allocation to the two procedures. The three subjects with <50 % excess weight loss were nonsmokers, two treated with SG, and one with RYGB. There was an excess weight loss of 76 ± 17 %, and BS was successful (i.e., >50 % excess weight loss) in 55 participants (95 %). In obese individuals, clinical and spirometric findings and all but serum and exhaled IL-8 biomarkers significantly improved or normalized (Table 1). Two subjects ceased cigarette consumption only.

One year after BS, AHR was abolished in 15 out of the 16 responsive individuals. In addition, three preoperative nonresponsive subjects became now responsive to mannitol. A univariate analysis (McNemar’s test) showed a significant reduction in AHR in obese individuals after BS (*p* < 0.001).

The results from the models fitted to all pre- and postoperative data failed to detect any significant interaction (all *p* values >0.2). The GEE analysis [31] showed that the period of evaluation before and after BS was the key independent factor associated with AHR improvement (OR, 4.7; 95 % CI, 1.4–15.4; *p* < 0.01). Cigarette smoking was not related to AHR. Likewise, postoperative changes in exhaled IL-8 and in AHR were not significant (OR, 1.09; 95 % CI, 1.0–1.2; *p* = 0.07).

## Discussion

This is the first prospective study that comprehensively assessed AHR to mannitol in morbidly obese, non-asthmatic adults, including smokers and nonsmokers. Two were the novel findings in our study. First, preoperative AHR to mannitol was abolished after BS in all but one individual and emerged in three new obese individuals. Second, the degrees of airway sensitivity (PD_15_) and reactivity (RDR) to mannitol were considerably altered in obese participants akin to patients with currently active asthma [26, 32]. The prevalence of AHR in obese nonsmokers was high when compared to what has been previously observed in young normal weighted adults [33]. Similarly, our results extend and complement previous studies using direct stimuli in normal weighted individuals [34, 35], obese adults with and without asthma [5], and overweight Chinese families of asthmatic adults [36].

The agents that act indirectly to induce AHR are considered more specific for identifying the presence of airway inflammation and more consistent with the diagnosis of asthma than direct stimuli [26]. Numerous hypotheses have been suggested to explain the relationships between obesity, asthma, and AHR, from dietary lifestyle through mechanisms of immunoregulation and inflammation [1, 37, 38]. Responsive obese nonsmokers had a higher likelihood of bronchial asthma and might therefore identify patients at high risk of asthma early before the expression of symptoms [10]. Notwithstanding, the mechanisms need to be probed further. From a clinical viewpoint, the significance of a positive response to mannitol is highly suggestive of active bronchial inflammation.

Before surgery, except for serum IL-8 values, obese individuals had higher biomarkers and lower adiponectin levels than control individuals, indicating more active systemic inflammation [39]. Serum adiponectin levels are reduced in obese subjects, and this is thought to induce systemic complications and augment airway inflammation [7, 19].

There were no differences in the rates of preoperative AHR between obese nonsmokers and smokers, and cigarette smoking was not related to AHR. Some smokers without clinical asthma can also have significant airway sensitivity to mannitol that is lost when smoking is stopped after a few weeks [40]. While the same mechanism of airway inflammation in obese nonsmokers could be invoked in smokers, it is known that smoking also recruits airway macrophages and neutrophils.

Effective weight loss promoted by BS was successfully achieved after surgery in the obese population and was associated to a practical abolition of AHR alone with lung function improvement, including that of most biomarkers and considerable reduction in comorbidities. Moreover, although the current study design did not preclude the role played by other factors that could influence postoperative evaluations potentially linked to changes in AHR, multivariate analysis suggested that BS was a key determinant of the significant AHR improvement observed in the obese cohort. The development of postoperative AHR in three new obese individuals (one nonsmoker and two smokers) and its persistence in one smoker suggest that aging and residual systemic inflammation may have also played an influential role.

The major strength of our study lies in its comprehensive design to assess clinical, functional, and biomarker outcomes in a cohort of asymptomatic, non-asthmatic, morbidly obese individuals with and without smoking habits, before and after surgery. There were, however, two shortcomings. First, the number of obese participants was not high due to the stringent design, and second, the indirect assessment of AHR in the current study was not compared with that made by direct stimuli because it was considered too aggressive in the setting of our study.

In conclusion, baseline preoperative AHR to mannitol in this cohort of asymptomatic morbidly obese candidates to BS is substantially high and associated to systemic inflammation; obese smokers had a similar rate of AHR but not associated to systemic inflammation. Our study provides new insights into the pathophysiology of AHR in obese individuals.
